# Prevalence and Predictors of Psychotropic Use in Children with High-Functioning ASDs

**DOI:** 10.1155/2013/384527

**Published:** 2013-05-16

**Authors:** Christopher Lopata, Jennifer A. Toomey, Jeffery D. Fox, Marcus L. Thomeer, Martin A. Volker, Gloria K. Lee

**Affiliations:** ^1^Institute for Autism Research, Canisius College, 2001 Main Street, Buffalo, NY 14208, USA; ^2^Summit Educational Resources, 150 Stahl Road, Getzville, NY 14068, USA; ^3^Autism Services Inc., 4444 Bryant Stratton Way, Williamsville, NY 14221, USA; ^4^Department of Counseling, School and Educational Psychology, University at Buffalo, State University of New York, 409 Baldy Hall, Buffalo, NY 14260-1000, USA

## Abstract

This study examined (1) the prevalence of psychotropic medication use for a sample of children with high-functioning autism spectrum disorders (HFASDs), (2) the extent to which psychotropic agents were linked to targeted symptoms, and (3) predictors of psychotropic use. A total of 115 children, ages 6–13, with HFASDs who were enrolled in psychosocial treatment trials were included in this study. Parents completed extensive background and rating forms prior to treatment that included data on demographic characteristics, child health, child medication use, and child ASD-related symptoms. Results indicated that 33% (*n* = 38) of the sample was taking psychotropic medication with the most common being stimulants (25%; *n* = 29), antidepressants (10%; *n* = 12), and neuroleptics (6%; *n* = 7). All children taking stimulants had target symptoms that were appropriate for stimulant medication, whereas 57% of those taking neuroleptics and 42% of those taking antidepressants did not have targeted symptoms consistent with the medication. Logistic regression for the major psychotropic drug categories indicated that lower IQ was a significant predictor of increased antidepressant and neuroleptic use. A higher level of ASD-related symptoms was related to the likelihood of stimulant use.

## 1. Introduction

Autism spectrum disorders (ASDs) are characterized by social and communicative impairments and circumscribed behaviors and interests [1]. Among the broader ASD population is a unique subgroup characterized as high functioning (HFASDs). Although this high-functioning subgroup exhibits core features of ASDs including impairments in social relatedness and stereotyped and repetitive behaviors and interests [1], they are differentiated from others with ASDs based on relative strengths in cognitive and formal language abilities [2]. Beyond core diagnostic symptoms, individuals with ASDs/HFASDs commonly exhibit a range of associated psychiatric symptoms that significantly disrupt daily functioning [3]. A number of studies have documented significantly elevated externalizing (e.g., ADHD, oppositional-defiant disorder, and conduct disorder) and internalizing symptoms in this population (e.g., depression and anxiety [4–7]).

 Although generally not approved to treat core ASD symptoms, psychotropic medications are frequently used to manage a range of cooccurring behavioral and psychiatric symptoms. Surveys have illustrated the common use of psychotropic medications in the broader ASD population, with prevalence rates ranging from 30% to 60% (e.g., [8, 9]). Aman and colleagues [10–12] completed several studies that examined psychotropic medication use and prescription patterns for individuals with ASDs. These studies documented rapidly changing prevalence, with rates increasing from 31% in 1995 to 46% in 2002 and 2003. The studies also found changes in the most commonly prescribed psychotropic medications, with neuroleptics being the most common in 1995 and antidepressants in 2002 and 2003. A more recent review found similar levels of psychotropic medication use, as well as the prominence of antidepressants among adolescents and adults with ASDs [8]. In a study that included children with ASDs, the prevalence of psychotropic use was approximately 39% and the most common type of agent was stimulants (17%) [9]. Beyond prevalence, researchers have attempted to better understand psychotropic medication use in ASDs by examining the relationship between subject characteristics and psychotropic agent use. These investigations have generally suggested that autism severity and intellectual impairment can be predictive of increased use of psychotropic medication in the broader ASD population (e.g., [9, 11]). 

Despite the suggestion that IQ and autism severity are predictive of psychotropic use, initial findings have suggested that individuals with HFASDs are also commonly prescribed psychotropic medications. Martin et al. [13] conducted the only identified study that examined psychotropic medication use among children, adolescents, and adults (*n* = 109) specifically with HFASDs. Results indicated that 55% of the sample was taking a psychotropic medication, with the most common being antidepressants (32%), stimulants (20%), and antipsychotics (17%). Despite the researchers' recommendation for studies of psychotropic use in HFASDs, theirs was the only study that focused exclusively on HFASDs. 

 Another critical consideration that has received little research attention is the match between medication type and target symptom [11]. Because target behavior guides the medication used [14], it is critical that these two are linked. Despite the critical importance of this alignment, almost nothing is known about the degree to which psychotropic agents are linked to target symptoms and studies are needed to examine the match between medications and target symptoms in this population [12].

The continual introduction of new psychotropic agents can affect medication trends [11] and warrant ongoing study. To date, studies of psychotropic use have generally used individuals with ASDs from broad age ranges and functional levels. Heterogeneous samples can confound conclusions about psychotropic medication use, as well as efficacy [15]. Studies of narrower age ranges are needed as age-related symptom presentation can potentially result in different prescription patterns [8]. Studies are also needed using samples of similar functional levels as target symptoms and psychotropic medication type appear to differ substantially for those with HFASDs compared to lower-functioning individuals with ASDs [13]. An additional limitation involves the fact that nearly all studies of psychotropic use in the ASD population have relied on parent-reported IQ levels and ASD severity which may underestimate impairments [11]. Further, IQ and ASD severity have been grossly defined categorically (mild, moderate, etc.) rather than by actual IQ or ASD symptom scores. This study extended the research by examining (1) the prevalence and type of psychotropic medication use, (2) the match between psychotropic agent and target symptom, and (3) predictors of use in a homogeneous group of children with HFASDs. IQ and ASD symptom data were collected directly using standardized measures.

## 2. Method

### 2.1. Participants

 Information was collected on 115 children, ages from 6 to 13 years, with HFASDs participating in separate psychosocial treatment trials over a 4-year period. All children in the psychosocial treatment trials met inclusion criteria using a multiple-gate screening procedure (see [16]). Criteria included a prior clinical diagnosis of an ASD, no indication of a current language delay, evidence of social interaction impairments (≥2) and restricted and repetitive behaviors and interests (≥1; per APA [1]), and a short-form IQ >70. In the first gate, parents submitted documentation of a prior diagnosis of Asperger's, autism, or PDD-NOS made by a licensed psychologist or psychiatrist and all relevant clinical and educational reports and records. Parents also completed a demographic form and developmental history. Once the required documents were received, the case was transferred to the second gate where two members of the senior research team independently reviewed the case using a standardized checklist composed of items indicating cognitive ability, current language levels, and DSM-IV-TR criteria (i.e., social interaction impairments and restricted, repetitive, and stereotyped patterns of behaviors or interests [1]). Each made an independent determination as to whether the documents supported the presence of a HFASD and clinical consensus between the two senior researchers was necessary to move the case to the third gate. In the third gate, participants completed an evaluation which included cognitive testing (i.e., Wechsler Intelligence Scale for Children-4th Edition [17] short-form) and informal observation of their social behaviors. The two senior researchers then reviewed the evaluation results and prior reports using the standardized checklist and independently made a determination as to whether results were consistent with a HFASD and met inclusion criteria. Consensus between the two researchers was required for inclusion. (For a description of the demographic characteristics of the sample, see Section 3.)

### 2.2. Instruments

#### 2.2.1. Medication and Demographic Surveys

As part of the prior studies' application processes, parents completed an extensive background packet that included sections on demographic characteristics, health-related issues/conditions (including seizures), and psychotropic medication use (along with other sections). In the medication section, parents reported on the children's current psychotropic medication(s) and identified the specific child symptoms each psychotropic agent was intended to treat. Current psychotropic medication indicated the medication(s) being taken at the time the survey was completed (there was no requirement that a medication had to have been taken for a minimum duration). 

#### 2.2.2. Wechsler Intelligence Scale for Children: 4th Edition (WISC-IV)

 IQ was evaluated using a 4-subtest short form of the WISC-IV [17] consisting of block design, similarities, vocabulary, and matrix reasoning subtests. Methods provided by Tellegen and Briggs [18] were used to calculate short-form reliability and validity coefficients. The short-form composite yielded an internal consistency estimate of .95 and correlated .92 with the Full Scale IQ.

#### 2.2.3. Behavior Assessment System for Children-2, Parent Rating Scales (Child (BASC-2 PRS-C) and Adolescent (BASC-2 PRS-A))—Developmental Social Disorders (DSD) Content Scale

The DSD content scale within the BASC-2 PRS-C and PRS-A [19] assesses ASD-related symptoms including deficits in social skills, communication, interests, and activities. Parents rate each item on a 4-point frequency scale (0 = never, 1 = sometimes, 2 = often, and 3 = almost always) with higher scores indicating more ASD symptoms. DSD coefficient alphas ranged from .82 to .85 for the age ranges included in this study across the PRS-C and PRS-A forms. Concurrent validity is supported in moderate correlations with other rating scales measuring similar skill deficits and adaptive areas (see [19]).

### 2.3. Procedures

The studies which generated these data were approved by the Institutional Review Board and conducted in accordance with the approved protocol. Written parental consent and child assent were obtained prior to the studies. As previously noted, the children with HFASDs were participating in psychosocial treatment trials and all met strict inclusion criteria. As part of the screening process, parents completed extensive background packets that included information on demographics, health-related issues/conditions, and medication use. They also completed the BASC-2 DSD prior to treatment. Parent-completed background packets and BASC-2 protocols were immediately reviewed upon return and omissions were corrected with parents. Each BASC-2 PRS protocol was scored using the BASC-2 ASSIST Plus computer scoring program. To increase accuracy in scoring, each BASC-2 protocol was independently scored by two research assistants. Data from the background packets and scores from the BASC-2 DSD were entered into the database by a research assistant and checked by a second research assistant.

Consistent with Esbensen et al. [8], psychotropic agents were classified into drug categories using the *Physicians' Desk Reference *[20]. The PDR was also used to determine whether the reported target symptom and psychotropic medication were linked (i.e., was the target symptom identified as an appropriate target for the specific psychotropic agent). Use of the PDR allowed for a consistent methodology for determining whether target symptoms and psychotropic agents were linked. Consistent with prior research (e.g., [8]), anticonvulsants were classified as mood stabilizers if there were no reported seizures.

## 3. Results

As previously noted, a total of 115 children, ages from 6 to 13 years, with HFASDs were included in this study. The sample was predominantly male (*n* = 104; 90.4%) and Caucasian (*n* = 102; 88.7%), with a mean age of 9.08 years (SD = 1.84). Average short-form IQ of the sample was 103.57 (SD = 14.84), and the average Developmental Social Disorders (DSD) score was *T* = 72.76 (SD = 8.57). Diagnostic breakdown (per prior clinical diagnosis) was Asperger's 55.7% (*n* = 64), PDD-NOS 31.3% (*n* = 36), and autism 13.0% (*n* = 15). 

Descriptive statistics were calculated to characterize the prevalence of psychotropic use and the link between psychotropic agent and target symptom (see Table 1). Results revealed that a total of 33% (*n* = 38) of the sample of children with HFASDs was taking a psychotropic agent at the time of the survey. Stimulants were taken by 25% of the sample, antidepressants by 10%, neuroleptics by 6%, and mood stabilizers by <1%. Of the subgroup of 38 children on a psychotropic medication, stimulants were taken by approximately 76%, antidepressants by 32%, neuroleptics by 18%, and mood stabilizers by 3%. Among the 38 children prescribed a psychotropic medication, 29% (*n* = 11) were taking more than one psychotropic agent.

Results of the comparisons examining the link between psychotropic medication and target symptom indicated that all children taking stimulants (*n* = 29) had target symptoms consistent with the stimulant medication, and the child taking the mood stabilizer (*n* = 1) had target symptoms consistent with the mood stabilizer medication. For antidepressants, 58% (*n* = 7) had target symptoms consistent with the antidepressant medication. Of the children taking neuroleptics, 43% (*n* = 3) had target symptoms consistent with the neuroleptic agent. 

Logistic regression (Table 2) was used to examine predictors of use for each prominent drug group separately (i.e., stimulants, antidepressants, and neuroleptics). Based on prior research studies, the independent variables included IQ and ASD symptoms (DSD). Model fit was established for each medication group using the Hosmer-Lemshow test (stimulants *χ* ^2^(8) = 8.91, *P* = .350; antidepressants *χ* ^2^(8) = 14.32, *P* = .074; neuroleptics *χ* ^2^(8) = 5.17, *P* = .739). For stimulants, ASD symptoms (DSD) were a significant positive predicator of use (*P* < .05). For both antidepressants and neuroleptics, IQ was a significant (negative) predictor of use (*P* < .05). No other characteristics were a significant predictor of psychotropic use. See Table 2 for detailed logistic regression results. (No analysis was done for mood stabilizers as only one child was taking a mood stabilizer medication.) 

## 4. Discussion

 To our knowledge, this was the first study to assess psychotropic medication prevalence, predictors of psychotropic use, and the link between psychotropic agent and target symptoms exclusively in children with HFASDs. At present, there is a need to examine psychotropic medication use in subpopulations with ASDs including groups defined by functional level (e.g., HFASDs [15]). This information is critical as children with HFASDs have needs that differ from lower-functioning children with autism, and they require medication treatments that target their unique symptoms and needs [13]. Many psychotropic medications offer a reduction of clinical and associated symptoms [21] which can enhance other forms of treatment and participation in social, educational, and family activities for individuals with ASDs/HFASDs [22]. The substantial increase in the pace of research involving psychotropic medications for this population [15], as well as the continual introduction of new agents highlights the need for ongoing study of prescription trends and the link between psychotropic agents and target symptoms [11, 12]. 

Results of this study indicated that one-third of the children with HFASDs in the sample were taking a psychotropic medication, and approximately 29% of the medicated group was taking more than one psychotropic agent. The overall 33% prevalence appears lower than that reported in other studies which utilized heterogeneous samples with ASDs (e.g., [8, 11]), as well as the 55% prevalence reported by Martin et al. [13] for children, adolescents, and adults with HFASDs. Interestingly, the psychotropic prevalence in this study was similar to the 39% prevalence recently reported by Rosenberg et al. [9] for 6 to 11 years olds with ASDs of variable functional levels. While the lower prevalence rate for children in the current study (and Rosenberg et al. [9]) may reflect greater apprehension to treat children with ASDs with psychotropic medications [22], it suggests that psychotropic agents are a common treatment for children with HFASDs. 

In addition, data from this study as well as Rosenberg et al. [9] also suggest that age may be an important factor in the type of psychotropic medication used. Studies that have included adolescents and/or adults with ASDs have most often found antidepressants and neuroleptics to be the most common psychotropic agents (e.g., [8, 12]). In contrast, stimulants were most common in the current sample (25%) of children with HFASDs, followed by antidepressants (10%), and neuroleptics (6%). Among the children taking psychotropic medications, stimulants were far more common than other types of psychotropic agents. Rosenberg et al. [9] also found that stimulants were most common (17%) for 6 to 11 years olds with ASDs in their study. This pattern of higher stimulant use is commensurate with the observation that ADHD-type behaviors are more common in children with HFASDs and that internalizing difficulties (e.g., depression and anxiety) become more pronounced during adolescence [23].

 To better understand psychotropic medication use in children with HFASDs, prominent characteristics identified in the literature (i.e., IQ and ASD symptoms) were examined to determine whether they were related to psychotropic use. IQ was found to be a significant predictor of antidepressant and neuroleptic use. Specifically, higher IQ scores were a significant predictor of less antidepressant and neuroleptic use. This finding is consistent with Rosenberg et al. [9] who found that lower IQ was a significant predictor of increased psychotropic use in ASDs. The unique aspect of the current finding is that IQ was still a significant predictor despite all participants having IQs >70. These findings differed from Martin et al. [13] who found that IQ was not associated with psychotropic medication use for their sample of children, adolescents, and adults with HFASDs. The discrepancy in findings may be a function of study differences (i.e., broader age range in Martin et al. [13]). The implication of IQ as a predictor of antidepressant and neuroleptic use may reflect more impaired general neurological functioning as cognitive level decreases, which increases use even in this group characterized by relative strength in cognitive ability. 

The other variable that was a significant predictor was ASD symptoms and this was restricted to stimulant use. Specifically, higher levels of ASD symptoms predicted increased stimulant use. The link between ASD symptoms and stimulant use was not necessarily surprising as children with HFASDs often exhibit symptoms that appear inattentive and “often receive an initial diagnosis of … ADHD” ([13], page 928). The finding that ASD severity only predicted stimulant use differs from other studies that have found that greater autism severity predicted increased use of various psychotropic agents in samples with ASDs (e.g., [11]). One possible explanation for the differences in findings involves the manner in which ASD severity was measured across studies. The current results were based on a standardized measure of ASD-related symptoms, whereas other studies have used parent-reported categories to determine ASD severity (autism versus Asperger's) which may account for the differences in findings. 

 This study also provided initial but needed insight into the extent to which target symptoms were linked to a specific psychotropic medication [12]. Findings indicated that all children taking stimulants (*n* = 29) and mood stabilizers (*n* = 1) had parent-identified symptom targets that were consistent with the specific medication. The overt nature of the behaviors targeted by stimulant medications may explain parents' ability to accurately identify the target behaviors. It is also possible that physicians clearly articulated these behaviors to parents when describing the medication target(s). The link between psychotropic agent and symptoms was less consistent for neuroleptics and antidepressants. Parents reported target symptoms that were consistent with the specific medication for only 43% of those taking neuroleptics and 58% of those taking antidepressants. One explanation is that physicians were using psychotropic medications for nonspecific targets. If true, this would represent a problematic prescription trend. It is also possible that parents have greater difficulty understanding or observing depressive symptoms and/or the behaviors targeted by neuroleptics. Alternatively, it is possible that physicians are failing to inform parents of these medication targets or they have difficulty articulating operational descriptors of the targets. Failure to convey such information is problematic as it is critical that parents can clearly identify the targets of psychotropic medications [22]. Ongoing research is needed to determine the cause of the discrepancy between parent-reported target symptoms and the symptoms the specific psychotropic medication is intended to treat. Because target symptoms were based on parent report, these initial findings should be interpreted with caution.

While this study was the first to examine psychotropic medication use, predictors of psychotropic use, and the link between target symptoms and specific medications in children with HFASDs and it had a number of strengths that addressed weaknesses in the research (e.g., structured screening, IQ test scores, ASD-symptom severity assessed using a standardized measure, narrowly defined group, etc.), several limitations warrant mention. One potential limitation involves sampling bias as the children with HFASDs were all beginning psychosocial treatment trials. Parents who pursued psychosocial treatment trials may differ from typical parents of children with HFASDs in their use of psychotropic medications. Another limitation involved the lack of data on the specialist who prescribed the medication [13]. Such information may provide insight into important prescription trends based on specialty. The current study also relied on parent reports for identification of psychotropic agents and target symptoms. Although use of parent reports is common in studies of psychotropic use in ASDs/HFASDs, future studies may seek to validate parent reports (e.g., review of prescriptions issued by physicians and/or physician reports). The sample was also small and largely male and Caucasian. Additionally, while the PDR allowed for a consistent methodology to examine the link between target symptoms and psychotropic agent, physicians often rely on a number of factors that affect their clinical decision making. Lastly, no statistical corrections were applied to the regression analyses so the findings should be considered as preliminary pending replication. Ongoing research is needed as individuals with HFASDs are distinct from lower-functioning individuals with autism, and they may require psychotropic medication treatment that targets different symptoms [13]. 

## 5. Conclusions

Results suggested that psychotropic agents were a relatively common treatment in this sample of children with HFASDs, with 33% taking at least one psychotropic medication. In contrast to other studies, the prominent type of psychotropic medication was stimulants (25%) which suggested potentially important age-related symptoms that clinicians should attend to in this high-functioning group. Antidepressant and neuroleptic medications were far less prevalent and only one child was taking a mood stabilizer. IQ played an important role in predicting antidepressant and neuroleptic use and it may serve as an important proxy for overall degree of impairment affecting the need for these psychotropic agents. Clinicians may also benefit from attention to the link between higher ASD-related symptoms and increased stimulant use given the tendency for many children with HFASDs to be viewed as exhibiting ADHD-type symptoms. Lastly, an important gap was observed between antidepressant and neuroleptic medication use and their identified target symptoms. Physicians may benefit from careful identification of the link between a specific psychotropic medication and target symptom(s), as well as ensure that parents clearly understand the symptom(s) targeted by the psychotropic agent. (For a comprehensive review of medication efficacy in ASDs, see [15, 24].)

## Figures and Tables

**Table 1:** Descriptive statistics for psychotropic medication use and link between target symptom and medication.

Medication class	*n*	% of total sample	% of medicated subjects	% positive link between symptom and medication (*n*)
Stimulants	29^a^	25.20%	76.32%^b^	100.00% (29)
Antidepressants	12^a^	10.43%	31.58%^b^	58.33% (7)
Neuroleptics	7^a^	6.09%	18.42%^b^	42.86% (3)
Mood stabilizers	1^a^	0.90%	2.63%^b^	100.00% (1)

Total sample size was *N* = 115.

Total number of individuals taking a psychotropic agent *n* = 38.

^a^Values do not total *n* = 38 due to 11 individuals taking more than one psychotropic agent.

^b^Values do not total 100% due to 11 individuals taking more than one psychotropic agent.

% of total sample = percentage of the total sample taking the specific medication class.

% of medicated subjects = percentage taking the medication class from the subgroup taking any psychotropic.

% Positive link between symptom and medication = percentage of individuals taking the specific medication class whose target symptoms were consistent with the symptoms addressed by the specific psychotropic medication (value in parentheses indicates the number of individuals whose target symptoms were linked to the specific psychotropic medication).

**Table 2:** Logistic regression results for the odds of taking a specific psychotropic medication class.

Model-medication class predictor	*B *	Wald	*P*	Odds ratio
Stimulants^a^				
IQ	−.024	2.554	.110	1.02
DSD (ASD symptoms)	.056	4.743	.029*	1.06
Antidepressants^b^				
IQ	−.043	3.892	.049*	1.04
DSD (ASD symptoms)	.039	1.218	.270	1.04
Neuroleptics^c^				
IQ	−.059	4.210	.040*	1.06
DSD (ASD symptoms)	−.002	.002	.963	1.00

*P < .05.

To aide interpretation, odds ratios for inverse relationships were transformed by 1/odds ratio.

Total sample size was *N* = 115.

DSD: developmental social disorders subscale from the BASC-2.

^a^Reference group is children with HFASDs not taking a stimulant medication.

^b^Reference group is children with HFASDs not taking an antidepressant medication.

^c^Reference group is children with HFASDs not taking a neuroleptic medication.
